# Progress in the Art of Farriery

**Published:** 1895-03

**Authors:** 


### PROGRESS IN THE ART OF FARRIERY.

A School of Anatomy and Scientific Horseshoeing has just been organized at Rochester, N. Y., under the direction of the local branch of the Master Horseshoers' National Association. Among the lecturers we note the names of the following well-known veterinarians: Albert Drinkwater, Emil Knight, J. D. Whyte, A. George Tegg, Leroy R. Webber and J. C. McKenzie.

These movements bode much good to the value, pleasure, and ownership of horses, and will greatly strengthen the veterinary profession as well as establish greater security and confidence to the owner of valuable animals.

A third School of Anatomy and Scientific Horseshoeing is announced at Detroit, Michigan. President Buckley, of the Master Horseshoers' National Association, has struck an unfortunate condition of affairs in Michigan, where the by-laws of the State Veterinary Organization deny their members the privilege of lecturing before such an organization. We believe this is a mistake that should be remedied by the members of that body. Later advices reach us that steps have already been taken to remedy this difficulty. We are sure that much good to all concerned will be derived by the diffusion of more technical knowledge among horseshoers, on whose judgment and knowledge depend so much the value and usefulness of the equine species. Aside from the great pleasure and satisfaction to be able to give scientific instruction as to the best method of shoeing a horse, and feeling secure that the same will be intelligently executed, no one save a veterinarian can so well appreciate the evils arising from the necessity of shoeing, and especially when this is ignorantly and defectively performed.

St. Louis is in the field with a school of anatomy and horseshoeing. The opening lecture was by Dr. J. M. Phillips, February 14, 1895. The attendance of horseshoers was large.

---

Philadelphia has added a third veterinarian to her corps of milk inspectors. The appointment was accorded to Dr. Chas. M. Earnest, V.M.D., graduate of the Veterinary Department of the University of Pennsylvania.

